# Role of endoscopic ultrasound for gallbladder disease

**DOI:** 10.1007/s10396-020-01030-w

**Published:** 2020-07-13

**Authors:** Kazunari Tanaka, Akio Katanuma, Tsuyoshi Hayashi, Toshifumi Kin, Kuniyuki Takahashi

**Affiliations:** Center for Gastroenterology, Teine Keijinkai Hospital, 1-40-1-12 Maeda, Teine-ku, Sapporo, 006-8555 Japan

**Keywords:** EUS, EUS-FNA, Gallbladder polyp, Gallbladder carcinoma

## Abstract

Endoscopic ultrasonography (EUS) has excellent spatial resolution and allows more detailed examination than abdominal ultrasonography (US) in terms of qualitative diagnosis of tumors and evaluation of tumor invasion depth. To understand the role of EUS in gallbladder disease, we need to understand the normal gallbladder wall structure and how to visualize it on EUS. In addition, gallbladder lesions can be classified into two broad categories: protuberant and wall-thickening lesions. Here, the features on EUS were outlined. We also outlined the current status of EUS-FNA for gallbladder lesions as there have been scattered reports of EUS-FNA in recent years.

## Introduction

Ultrasonography (US) is widely performed as a screening test for gallbladder lesions as it is less invasive and can be done easily. Computed tomography (CT) or magnetic resonance imaging (MRI) is often used for further evaluation when clinical questions persist after US has been performed. CT is suboptimal for spatial resolution and hence limited in its ability to provide differential diagnosis of gallbladder lesions. However, it is useful for diagnosis of the presence of certain large gallbladder lesions and their progression. In cases where malignancy is suspected based on other tests, a qualitative diagnosis by MRI is useful. It is also possible to understand the overall picture of the disease by magnetic resonance cholangiopancreatography. EUS has a high spatial resolution and allows for a more detailed examination of the gallbladder because it can approach and examine the organ at a closer range than US. This makes it possible to make a qualitative diagnosis of lesions and evaluate tumor invasion depth with EUS. There have also been recent studies on EUS fine-needle aspiration (FNA) in gallbladder disease. Thus, EUS-FNA may potentially augment difficulties in the pathological examination of gallbladder lesions.

Herein, we describe the current applications of EUS-FNA for gallbladder lesions.

### 1. Normal anatomy of the gallbladder wall

**Fig. 1:**
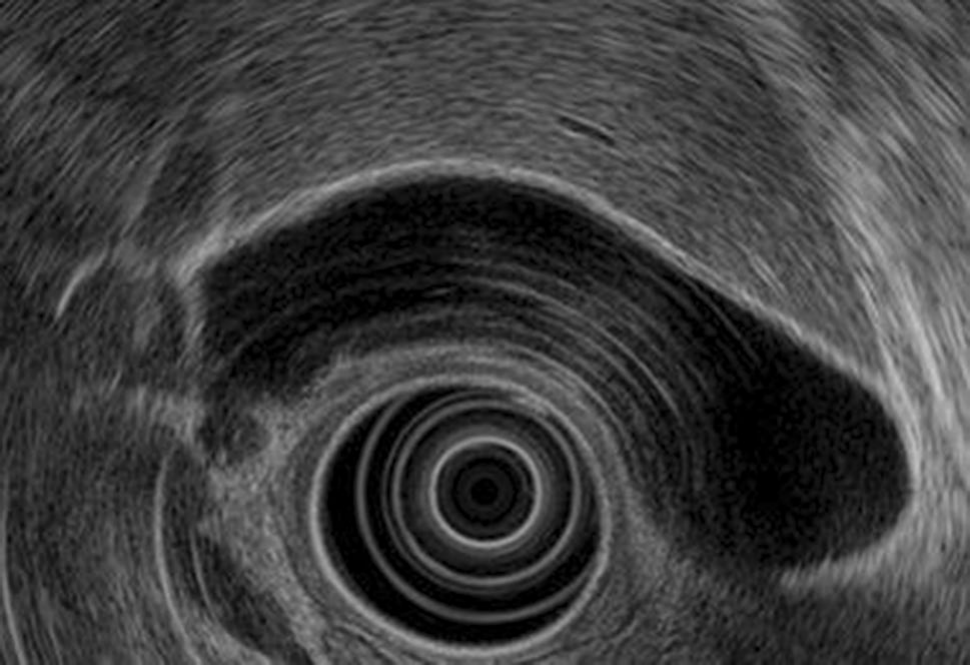
Normal gallbladder. The gallbladder wall is divided into an inner low echoic layer and an outer high echoic layer

In US, the gallbladder wall is visualized as two layers, a hypoechoic inner layer and a hyperechoic outer layer, which correspond to the mucosa through the shallow and deep subserosal layers, respectively [1] (Fig. 1). Normally, the gallbladder wall is at most 3 mm thick with a smooth luminal surface. A gallbladder wall measuring ≥ 4 mm is considered to be thickened.

### 2. Methods for visualization of gallbladder lesions by EUS

There are two types of EUS scopes, radial scanning and convex array. These devices provide different images and are therefore used in various visualization methods. Kaneko et al. [2] performed a prospective comparative study on the differences in visualization between these two devices in the examination of the pancreaticobiliary region.

With regard to gallbladder long-axis visualizing capability, convex array EUS was inferior to radial scanning EUS. However, there was no significant difference in the lesion imaging and new lesion imaging between the two groups. Hence, in settings where both devices are available for use, the features of each device should be clearly understood, and proper use should be implemented according to the patient’s medical condition.

To minimize oversight in testing, confirmation using different modalities such as magnetic resonance cholangiopancreatography is important before EUS. As the gallbladder structure and position vary from patient to patient, the gallbladder curvature and the positions of lesions should be verified in advance, preventing oversight during EUS. The position of the gallbladder fundus, in particular, varies largely between individuals, and caution is necessary as failure to ascertain the overall gallbladder structure before performing the examination may result in lesion oversight and inability to obtain accurate observations.

The key points for gallbladder examinations for each type of EUS are presented below.

#### i) Radial scan type

**Fig. 2:**
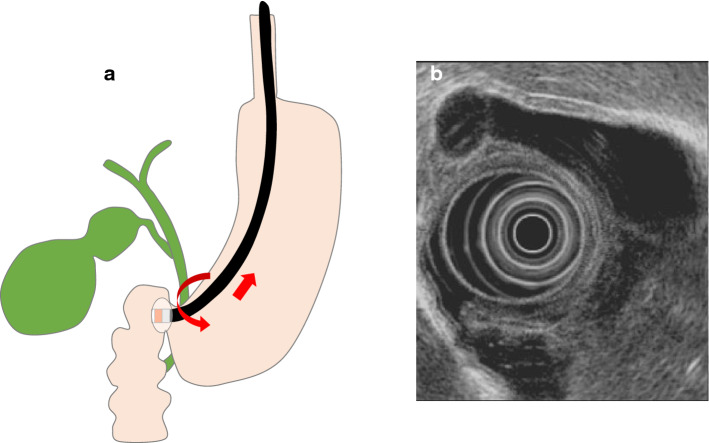
Radial scan type (short-scope position). **a** Short-scope position. **b** EUS image of the gallbladder in the short-scope position

**Fig. 3:**
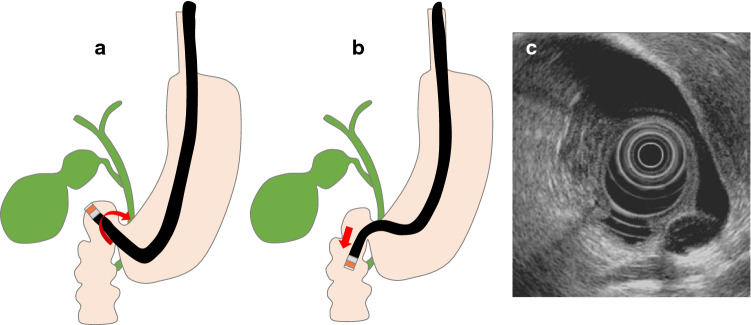
Radial scan type (long-scope position). **a** Long-scope position in the duodenum bulb. **b** Clockwise rotation causes the scope to advance in the direction of the descending duodenum

For a gastric scan, after observing the pancreas, advance the scope into the descending duodenum and stretch the scope into the short scope position. Inflate the balloon slightly, identify the bile ducts, withdraw while rotating counterclockwise, and identify the cystic duct junction. Inflate the balloon further, withdraw the scope, and examine from the cystic duct to the neck of the gallbladder (Fig. 2a). Depending on the patient, it may be possible to examine the whole gallbladder, including the fundus (Fig. 2b). In cases where the whole gallbladder cannot be examined in the short scope position, in the duodenal bulb, press the scope tip against the superior duodenal flexure, tilt the scope upward, and lightly advance it assuming a long scope position (Fig. 3a). Clockwise rotation causes the scope to advance in the direction of the descending duodenum (Fig. 3b). Visualize the cystic duct junction, and successively examine the cystic duct and the neck, body, and fundus of the gallbladder (Fig. 3c). During this procedure, the short scope position and gallbladder direction are opposite. In transgastric scanning, observation may be possible by either withdrawing the scope with the balloon inflated from the short scope position or pressing the scope against the pyloric ring with the balloon inflated and assuming the long scope position.

#### ii) Convex array

**Fig. 4:**
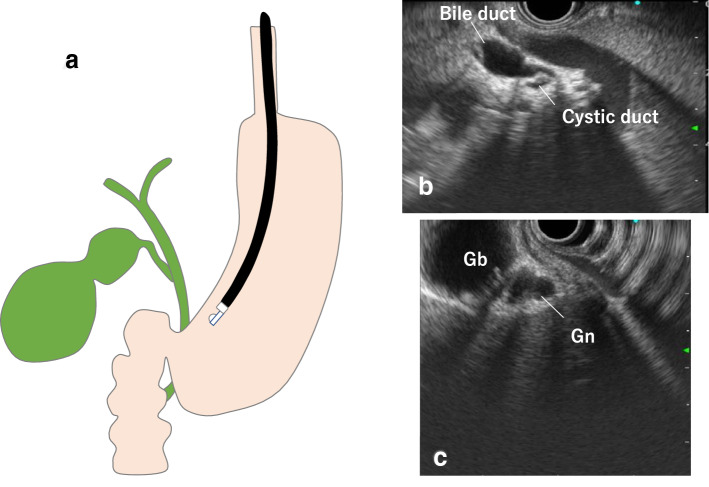
Convex array (transgastric scanning). **a** EUS image of the cystic duct junction. **b** EUS image of the gallbladder neck and body. Gn: neck of gallbladder, Gb: body of gallbladder

When examining the gallbladder by transgastric scanning, it is easier to identify the bile ducts using the portal vein as the starting point. By following the intrahepatic portal vein of the left lobe of the liver and identifying the hilar portal vein, the hilar hepatic ducts can be visualized in the deep part of the portal vein (Fig. 4a).

Accordingly, visualization of the cystic duct junction is occasionally possible by continuing to advance the scope, and visualization from the cystic duct to the gallbladder neck is occasionally possible by rotating the scope (Fig. 4b). However, as the direction of rotation at this point varies with each patient, visualization should be performed while carefully following the cystic duct (Fig. 4c). Note that examination of the entire gallbladder from within the stomach is not always possible.

**Fig. 5:**
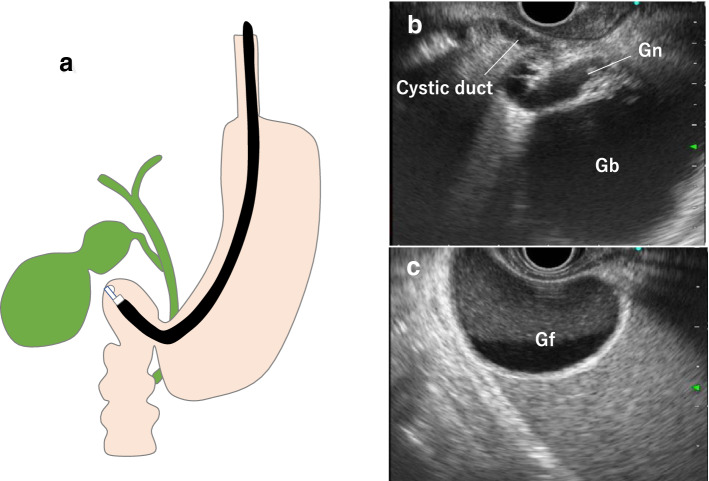
Convex arrayed (duodenal bulb scanning). **a** EUS image of the cystic duct and gallbladder neck and body. **b** Fundus of gallbladder. Gf: fundus of gallbladder

In duodenal bulb scanning, the hilar hepatic ducts are identified by visualizing the portal vein and tilting the scope downward while withdrawing it in counterclockwise rotation (Fig. 5a). The cystic duct junction can be recognized through this process (Fig. 5b). By rotating the scope while following the cystic duct, it is possible to visualize the whole gallbladder from the neck to the fundus (Fig. 5c). As the direction of rotation at this point also varies from patient to patient, it is important to make a thorough observation including the gallbladder fundus by visualizing successively from the cystic duct toward the gallbladder neck.

#### iii) Contrast-enhanced harmonic EUS

**Fig. 6:**
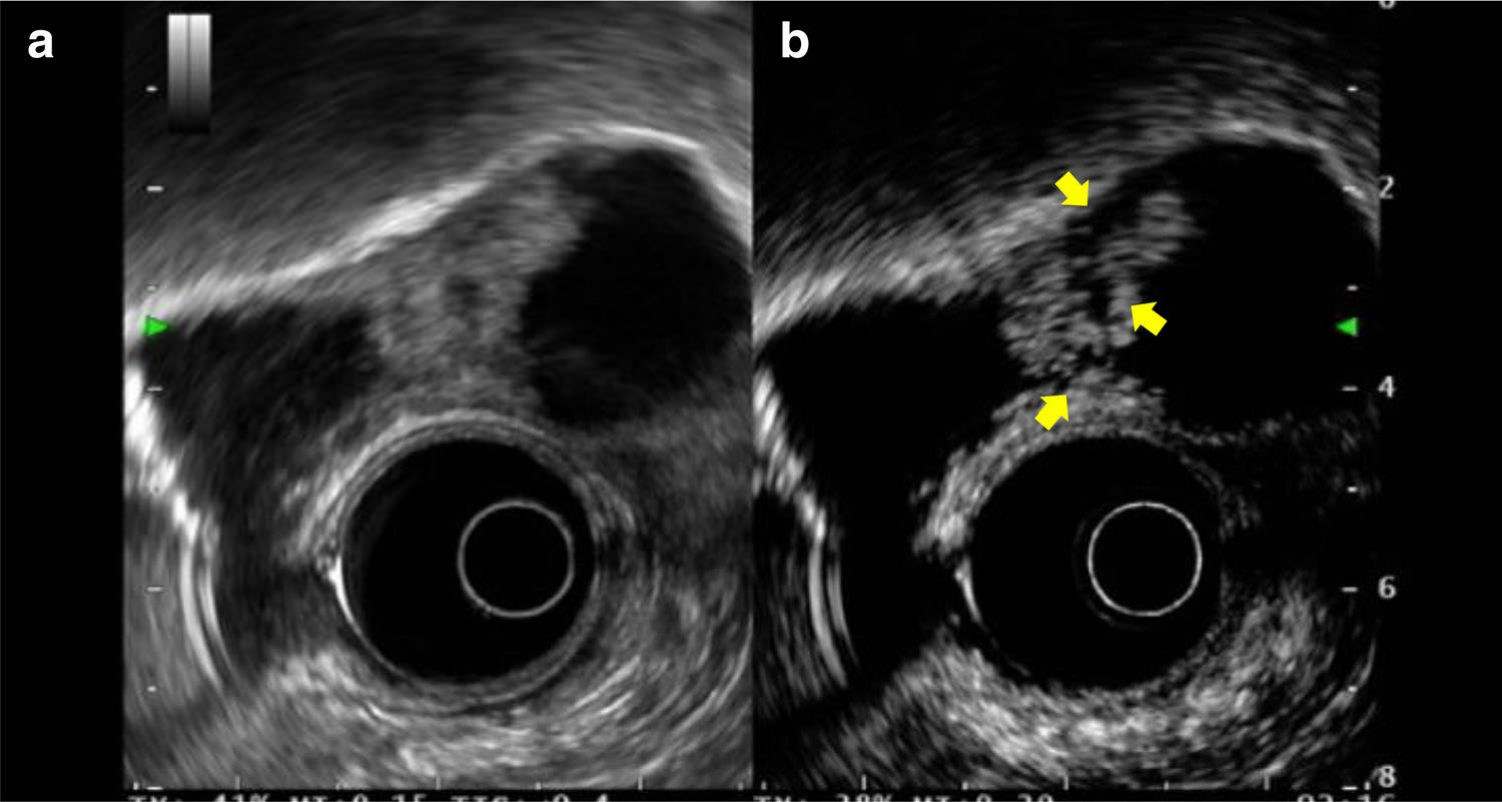
Contrast EUS for gallbladder carcinoma. **a** Conventional EUS demonstrates a hypoechoic mass in the gallbladder. **b** Contrast-enhanced harmonic EUS indicates that the area has perfusion defects (arrow)

Although contrast-enhanced harmonic EUS for gallbladder disease is not covered by health insurance, Choi et al. [3] have reported that the presence of irregular intratumoral vessels and a perfusion defect on contrast EUS can diagnose gallbladder cancer in gallbladder polyps measuring at least 10 mm with a sensitivity and specificity of 93.5 and 93.2%, respectively (Fig. 6). Imazu et al. [4] also reported that inhomogeneously enhanced patterns were observed in contrast EUS. However, further accumulation of knowledge is desired as there has been apparently no large-scale study on contrast-enhanced harmonic EUS in gallbladder diseases to date.

### 3. Differential diagnosis of gallbladder lesions

Gallbladder lesions are broadly divided into protuberant and wall-thickening lesions. Protuberant lesion is an inclusive category encompassing a variety of diseases, both epithelial and non-epithelial, as well as benign and malignant diseases. It is a generic term for lesions that have the specific morphological feature of forming a protuberance localized to the luminal side of the gallbladder [5]. In differentiating protuberant gallbladder lesions, the classification of benign protuberant lesions by Christensen et al. is used [6]. However, from a clinical perspective, the significance of treating lesions collectively as gallbladder polyps before a definitive diagnosis lies in the early detection of malignant disease from these lesions. Therefore, protuberant gallbladder lesions are first divided into neoplastic and non-neoplastic lesions. Differential diagnoses such as adenomas or carcinomas for neoplastic lesions and cholesterol polyps, hyperplastic polyps, and gallbladder adenomyomatosis for non-neoplastic lesions are based on size, pedunculation, morphology, surface characteristics, and internal echo. On the other hand, wall-thickening lesions denote lesions in which the gallbladder wall is diffusely thickened. Differential diagnosis is made with reference to the extent of wall thickening, surface structure, and presence or absence of Rokitansky–Aschoff sinuses (RAS).

Ultrasonographic features of gallbladder protuberant lesions and gallbladder wall-thickening lesions are summarized in Tables. 1 and 2, respectively.

**Table 1:** Ultrasonographic features of gallbladder protuberant lesions

	Form	Surface	Internal echo
Cholesterol polyp	・Morular or oval	・Granular	・Rough or granular ・Highly echogenic punctiform foci
Hyperplastic polyp	・Papillated or lobulated	・Smooth	・Low echogenicity ・Uniform low echogenicity
Inflammatory polyp Fibrous polyp Granulomatous polyp	・Ovla or lobulated	・Smooth ・Hyperechoic polyp surface border	・Anechoic spots ・Uniform low echogenicity
Adenomyomatosis	・Sessile or oval	・Smooth	・Anechoic spots ・Comet tail artifact ・Uniform low echogenicity
Adenoma	・Oval	・Smooth or nodular	・Solid echogenicity ・Multiple microcystic spaces
Gallbladder carcinoma	・Oval or irregular	・Smooth or irregular	・Uniform internal echo ・Dense solid echo

**Table 2.** Ultrasonographic feature of gallbladder wall thickened lesions

	Surface	Internal echo
Adenomyomatosis	・Smooth or irregular	・Cystic anechoic spots ・Comet tail artifacts
Xanthogranulomatous cholecystitis	・Smooth	・Mixed hyperechoic and hypoechoic echotexture
Anomalous pancreaticobiliary junction	・Smooth	・Uniform low echogenicity
Gallbladder carcinoma	・Irregular or papillated	・Uneven hypoechogenicity

### 4. Protuberant lesions

#### i) Non-neoplastic lesions (gallbladder polyps)

Gallbladder polyps are small, localized, raised lesions observed on the mucosal surface of the gallbladder. Histopathologically diverse diseases are included, whether benign or malignant, neoplastic or non-neoplastic, and epithelial or non-epithelial. In daily clinical practice, benign lesions measuring < 2 cm are usually detected [7]. Most gallbladder polyps are asymptomatic and discovered incidentally during medical or comprehensive health examinations. The prevalence rate is reported to be within 4.2–9.5% in East Asia [8–11] and 3–7% in Western countries [12].

**Fig. 7:**
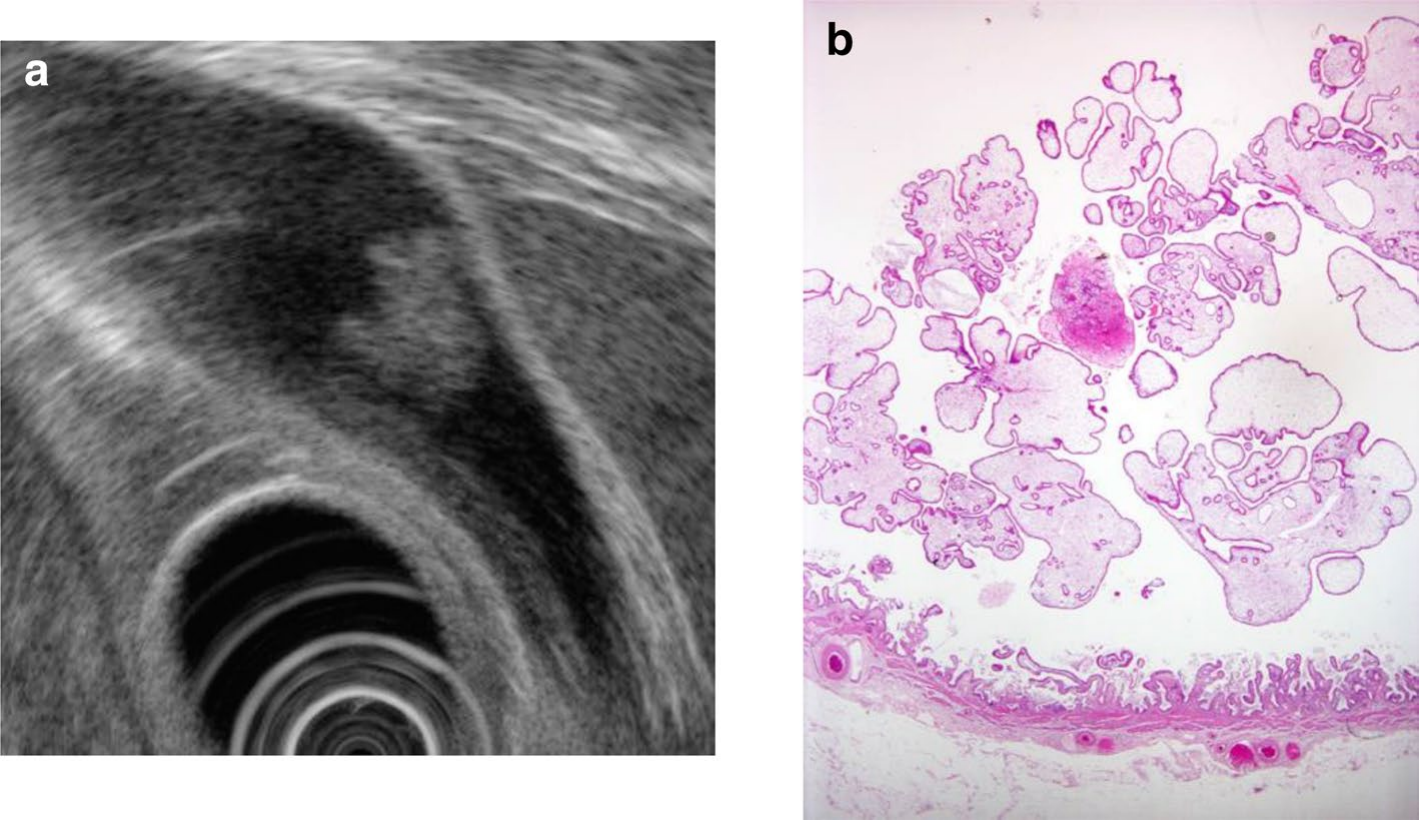
Cholesterol polyp. **a** This polyp has a granular surface and morular morphology. The internal echo is rough or granular. **b** Polypoid lesion with non-neoplastic epithelium and abundant stroma

The main types of gallbladder polyp are as follows:

A. Cholesterol polyps: These are the most common gallbladder polyps and comprise 62.8% of all gallbladder polyps. Although multiple polyps measuring ≤ 10 mm are highly likely to be cholesterol polyps, [5] caution is necessary as 5% of polyps are cancerous even if they measure ≤ 10 mm [13]. The characteristic findings on EUS are a deeply notched granular surface and morular morphology. The internal echo is rough or granular, and highly echogenic punctiform foci reflecting cholesterolosis are visible [14] (Fig. 7). Peduncles are thin and frequently unobserved even on EUS.

**Fig. 8:**
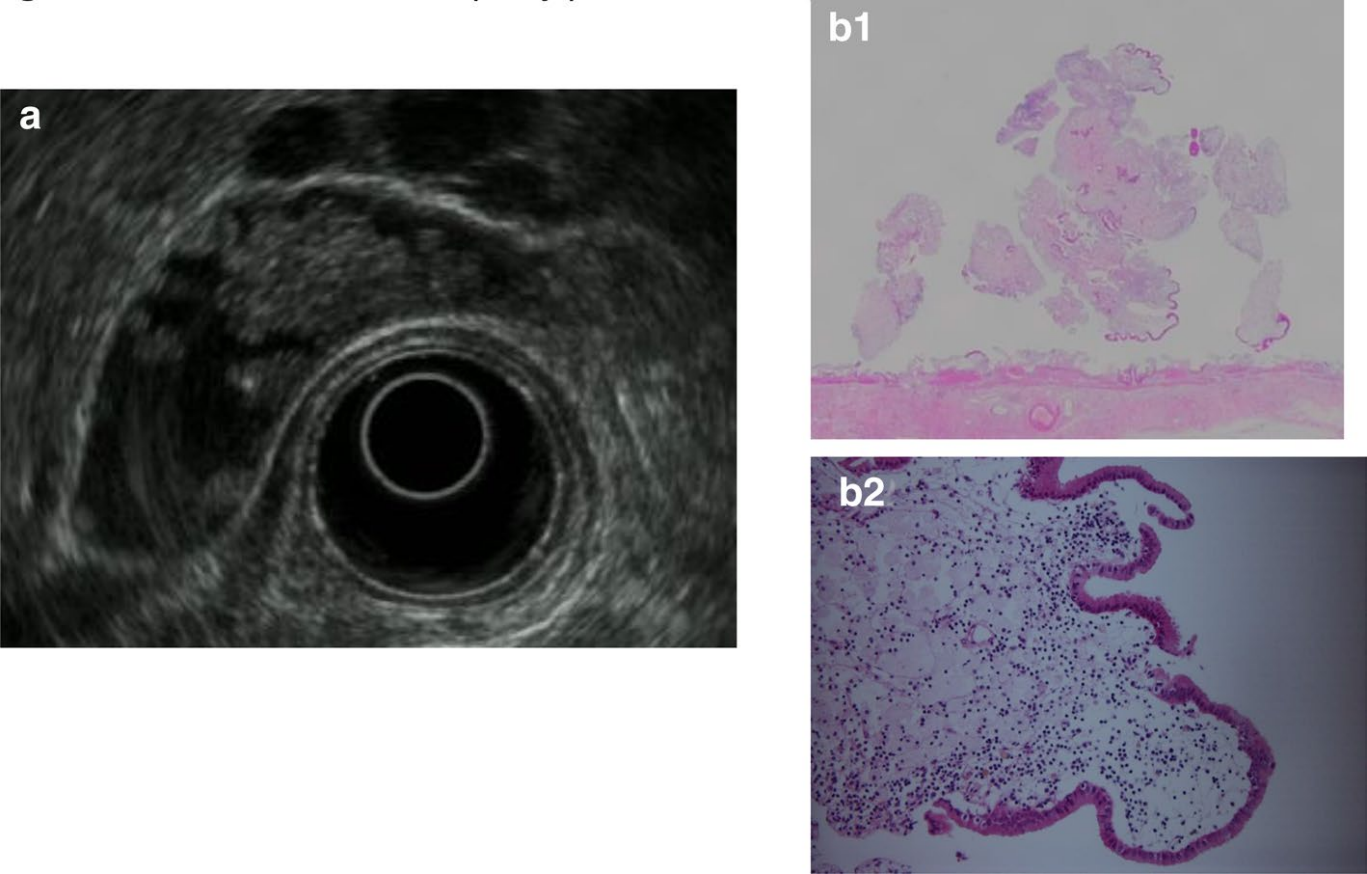
Cholesterol polyp resembling early gallbladder carcinoma. **a** EUS image of a solid internal echogenicity polyp without echogenic punctiform foci. **b** (1, 2) Photomicrograph demonstrating an aggregation of foamy cells under the epithelium

When polyps reach ≥ 10 mm, epithelial hyperplastic changes are reflected as lobulation, and internal echo decreases, making differentiation from adenoma and early gallbladder cancer difficult in some cases and necessitating caution (Fig. 8).

**Fig. 9:**
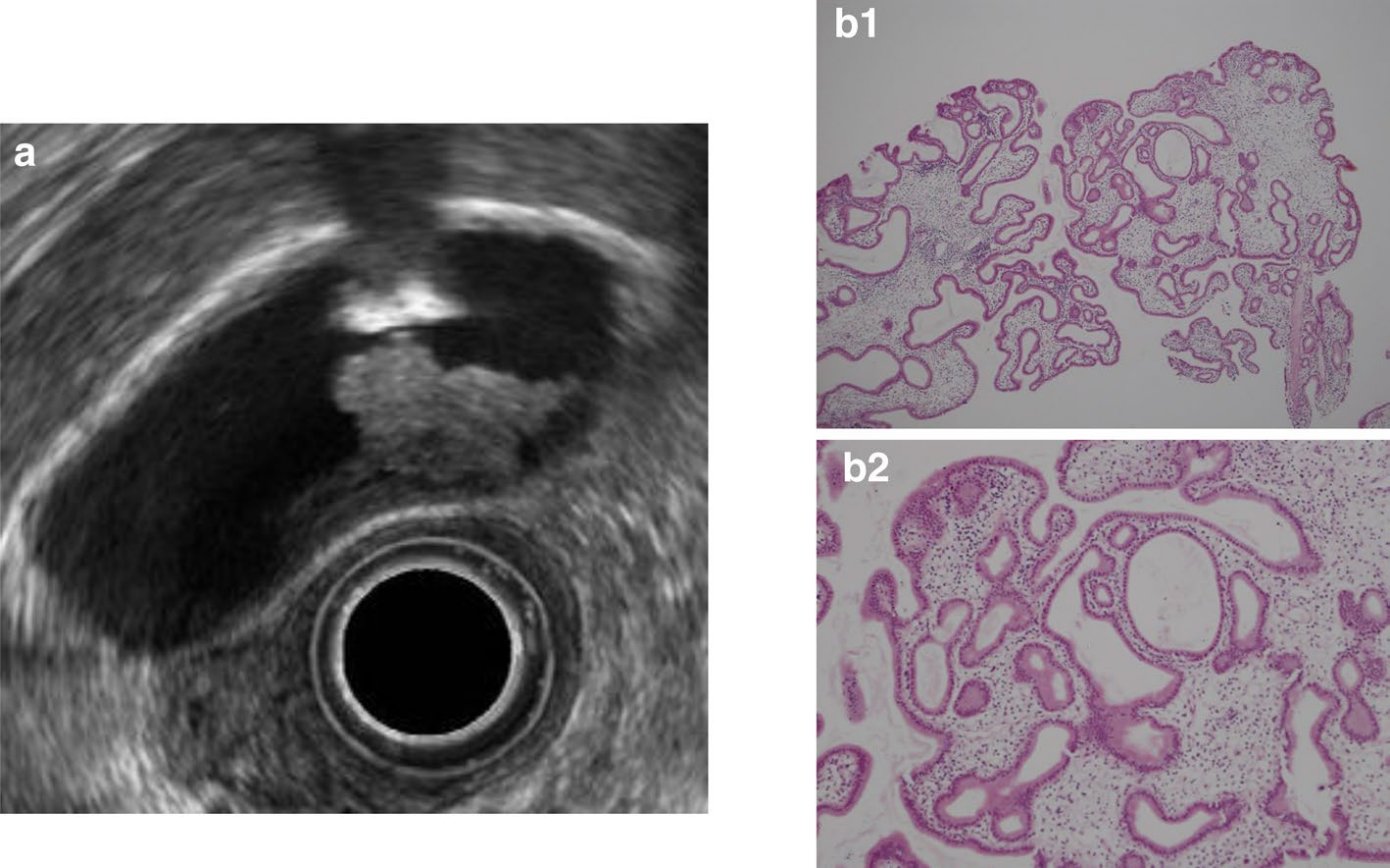
Gallbladder hyperplastic polyp. **a** EUS image of a pedunculated, lobulated, solid internal echogenicity polyp. **b** (1, 2) The polyp consists of duct glands similar to the pyloric gland

B. Hyperplastic polyps: Hyperplastic polyps are classified as proper epithelial or metaplastic epithelial polyps, and they frequently multiply. The proper epithelial type occurs singly, measures ≥ 10 mm, is papillated to lobulated, and shows relative internal uniformity. If accompanied by cholesterolosis, internal punctiform echogenic foci are observed, which complicates differentiation from cholesterol polyps (Fig. 9).

C. Inflammatory, fibrous, and granulomatous polyps: Whether to treat inflammatory, fibrous, and granulomatous polyps as distinct or similar remains controversial. Inflammatory polyps are relatively rare, comprising 1.4–12% of gallbladder polyps [15–18]. These polyps, which result from hyperplasia of edematous loose connective tissues, are internally hypoechoic and occasionally accompanied by inflammatory thickening of the gallbladder wall.

**Fig. 10:**
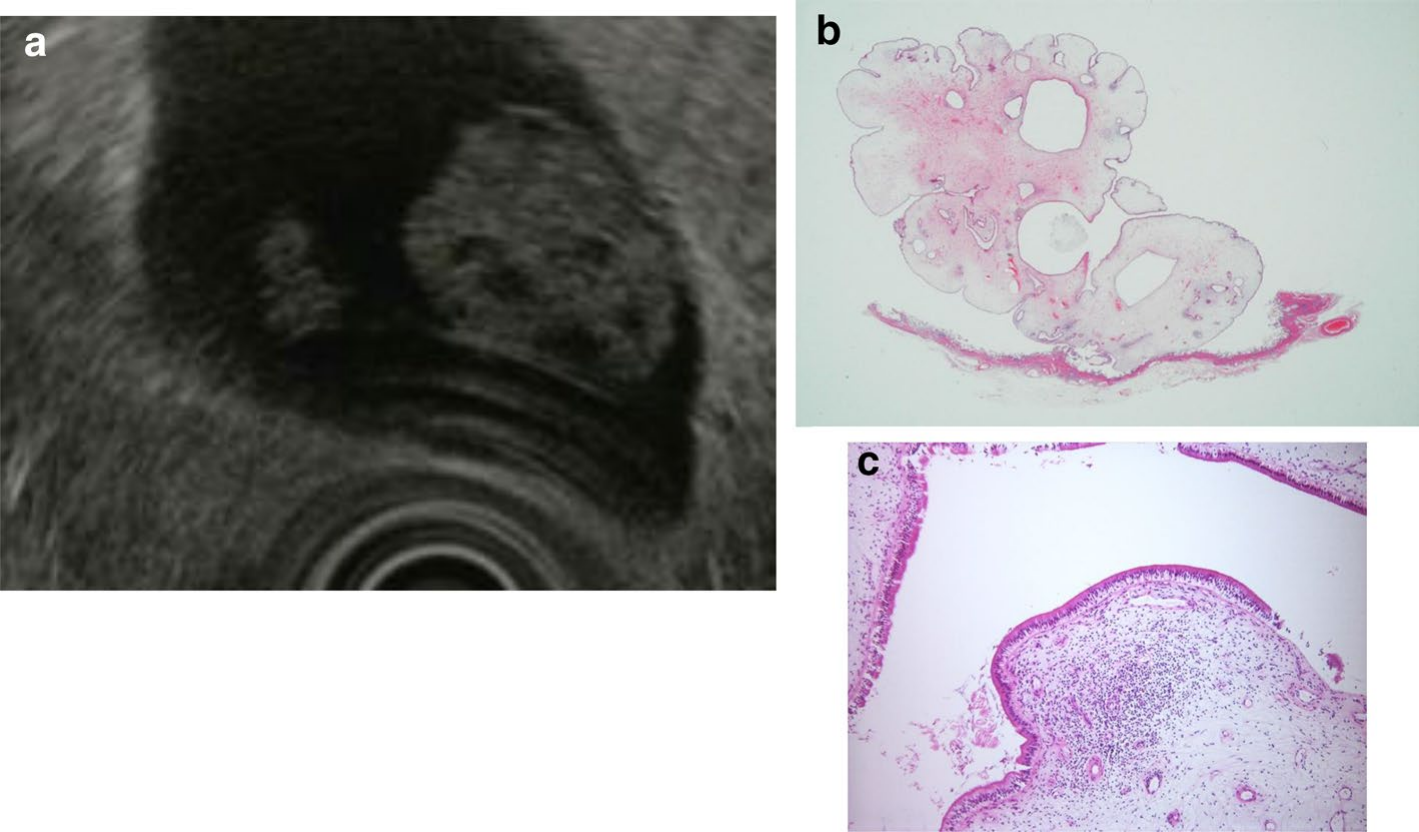
Gallbladder inflammatory polyp. **a** EUS image showing a pedunculated, smooth surface polyp with an anechoic area. **b** (1, 2) The stroma consists of edematous and coarse fibrous connective tissue. The surface iconsists of simple columnar epithelium

**Fig. 11:**
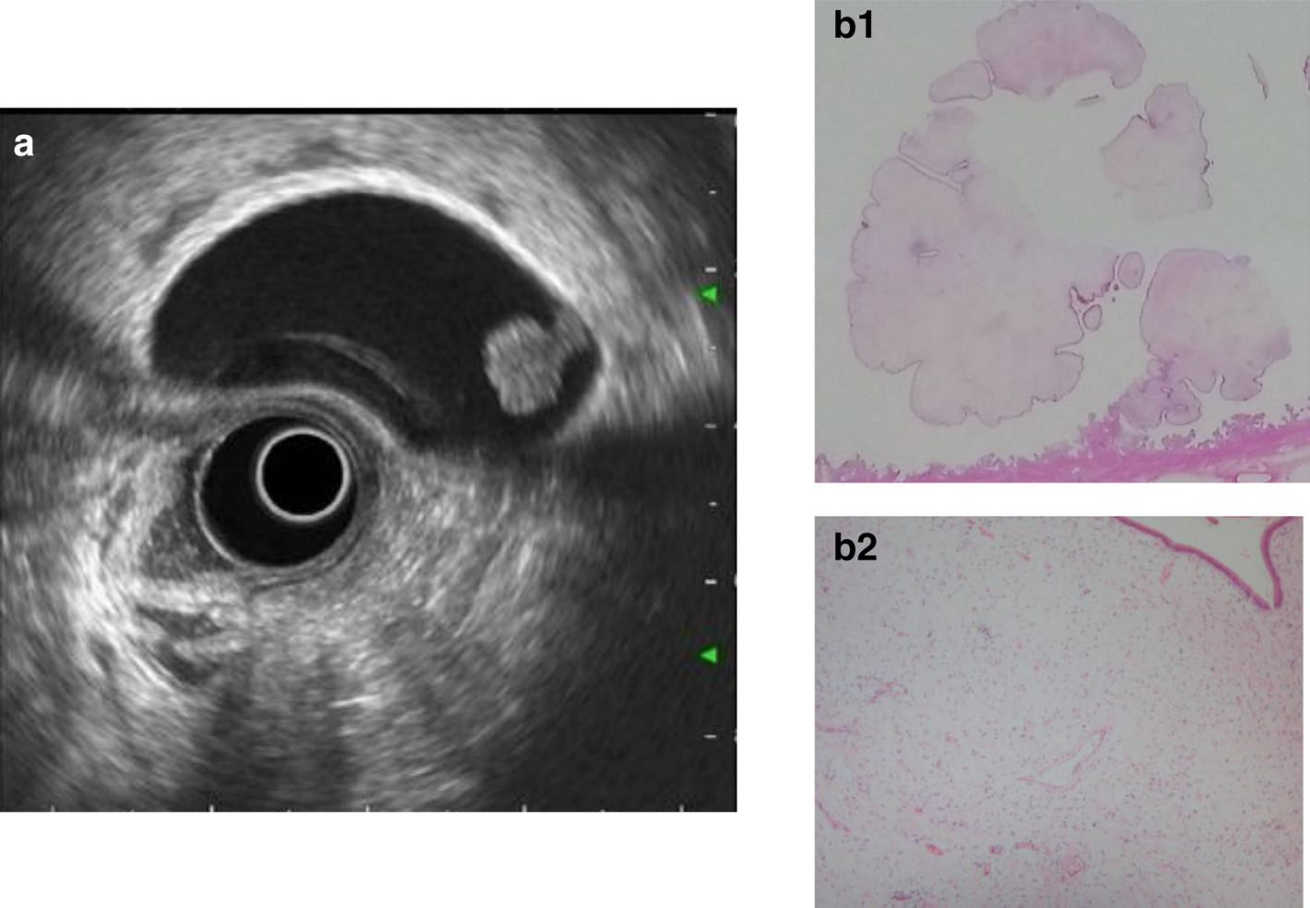
Gallbladder fibrous polyp. **a** EUS image showing a pedunculated, smooth surface, uniformal echogenicity hypoechoic polyp. **b** (1, 2) The stroma consists of edematous and coarse fibrous connective tissue. The surface consists of simple columnar epithelium

The characteristic EUS findings are internal anechoic spots with hyperechoic polyp surface borders. These findings appear to occur because of the difference in the acoustic features between the single surface layer of the columnar epithelium and the edematous stroma [19] (Fig. 10). Fibrous polyps are made up of connective tissue composed of fibroblasts, fibrocytes, and collagen fibers, and imaging findings resemble those of inflammatory polyps (Fig. 11). Granulomatous polyps, which are formed from inflammatory granulation tissue, lack a surface epithelium and have a high rate of comorbidity with acute cholecystitis and gallstones.

#### ii) Neoplastic lesions

**Fig. 12:**
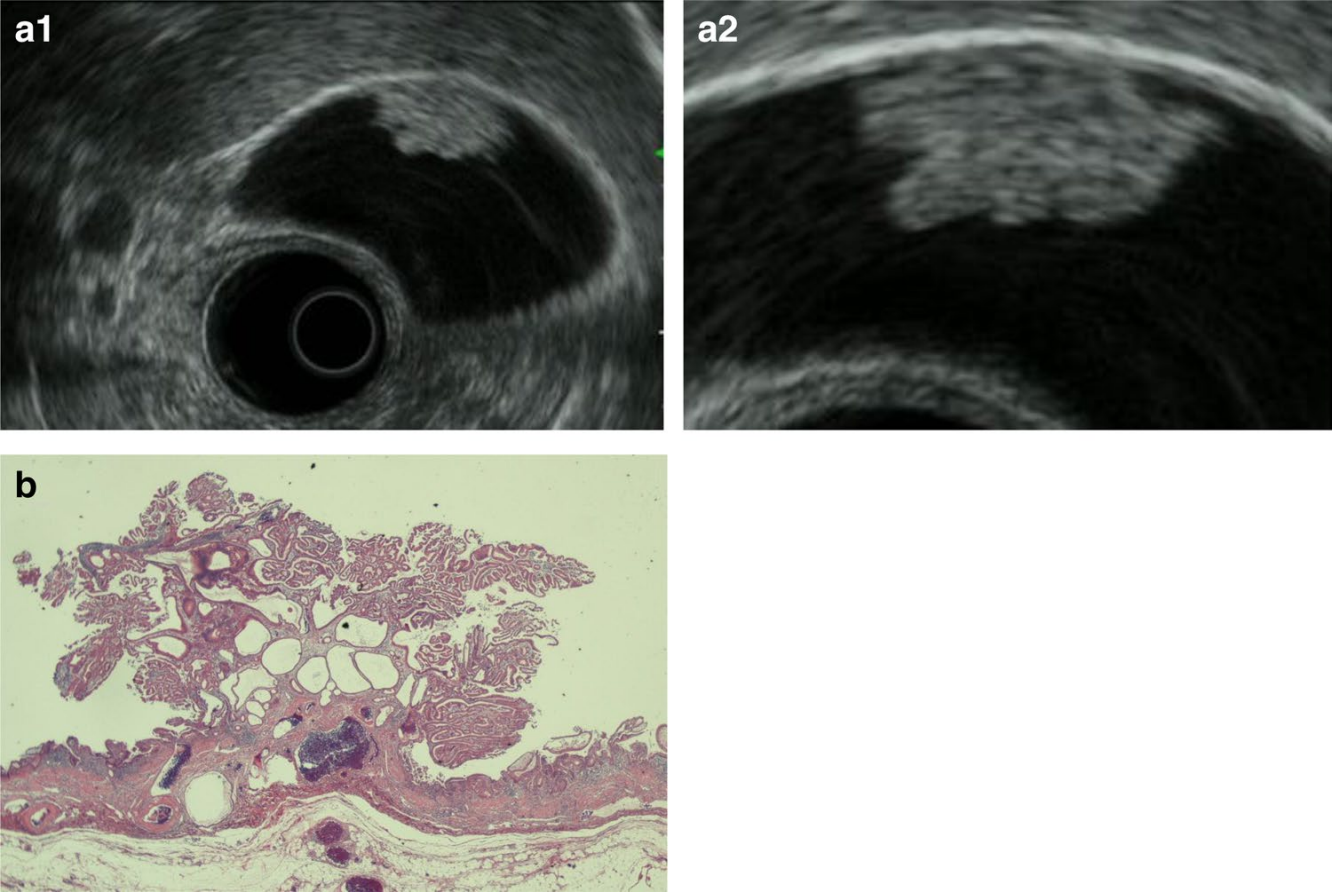
Gallbladder adenoma. **a** (1, 2) EUS image showing a relatively smooth surface, solid internal echogenicity polyp with multiple microcystic spaces. **b** Photomicrograph imaging of the gallbladder adenoma

A. Adenomas: Adenomas are classified as tubular or papillary. Tubular adenomas, of which pyloric adenomas are common, are pedunculated to subpedunculated and oval. The features on EUS are a relatively smooth or nodular surface, solid internal echogenicity, and the presence of enlarged neoplastic glandular ducts observed as multiple microcystic spaces [20] (Fig. 12). Papillary adenomas are predominantly of the proper epithelial type with a low solid echo and must be differentiated from hyperplastic polyps. Differentiation between adenomas and adenocarcinomas based on imaging is considered difficult.

**Fig. 13:**
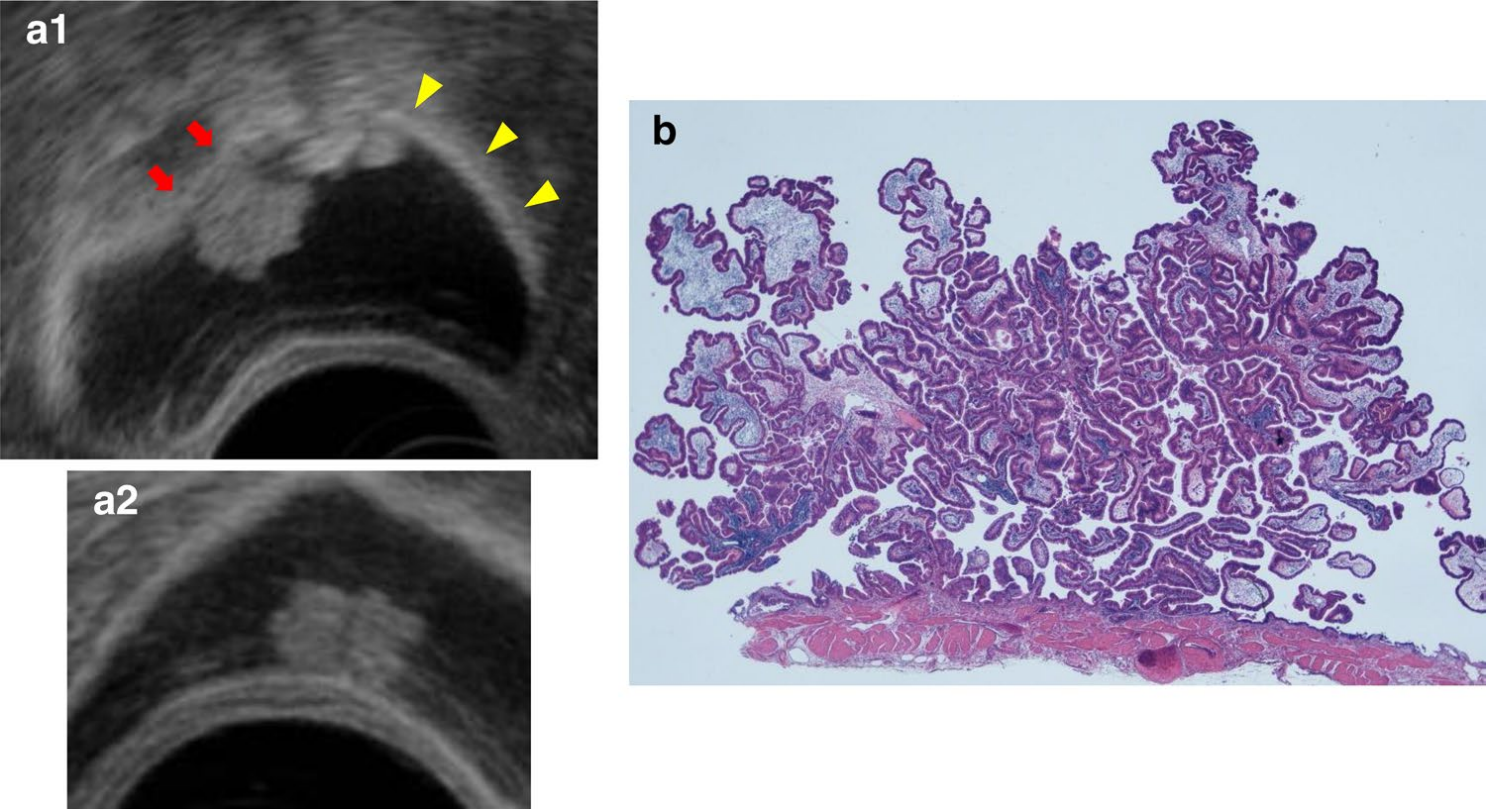
Early gallbladder carcinoma. **a** (1, 2) EUS image: A homogenously hypoechoic protruding lesion with a granular surface is seen. The outer layer of the gallbladder is well preserved (arrow, retained hyperechoic outer layer; arrowhead, normal hyperechoic outer layer). **b** Photomicrograph: A pedunculated polypoid lesion was diagnosed as well-differentiated adenocarcinoma. It was invading into but not through the muscularis layer

**Fig. 14:**
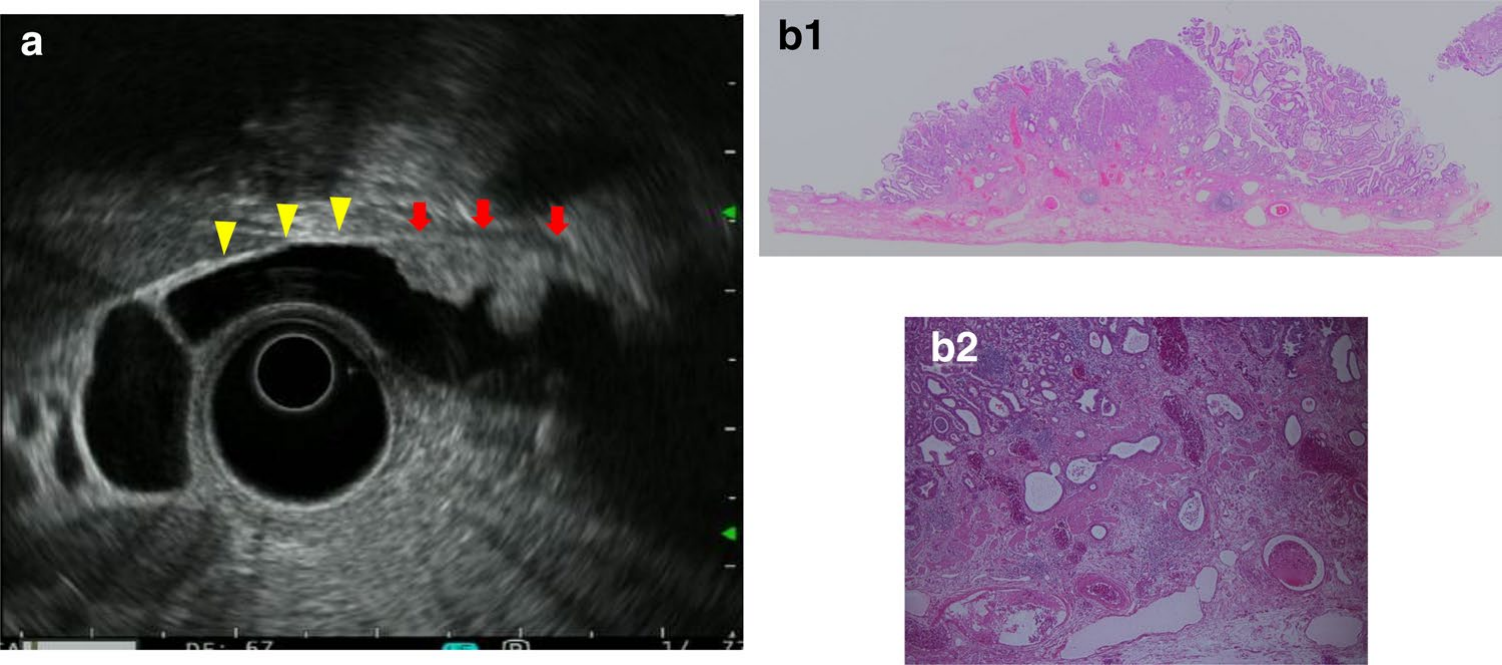
Advanced gallbladder carcinoma. **a** EUS imaging of a sessile elevated lesion with thinning of the hyperechoic outer layer (arrow, thinning of the hyperechoic outer layer; arrowhead, normal hyperechoic outer layer). **b** (1, 2) Photomicrograph: Gallbladder carcinoma with SS depth invasion

B. Gallbladder carcinoma (protuberant type): Gallbladder carcinoma is considered in cases of diffuse or localized irregular thickening of the gallbladder wall in which an irregular mucous membrane surface and a loss of uniformity in the inner hypoechoic layer are observed. Ultrasound imaging findings are classified as protuberant (peduncular/sessile), wall thickening, or both types. Of the protuberant type, peduncular lesions (type Ip) often show morphological resemblance to adenomas (Fig. 13), uniform internal echo, and dense solid echo. Adenocarcinomas are common among type Ip, whereas sessile lesions (types Is and IIa) are frequently accompanied by associated neighboring IIa and flat lesions. Because the layered structure can be examined in detail by EUS, type Is lesions with a deep hypoechoic area or thinning of the hyperechoic outer layer (Fig. 14) can be diagnosed as gallbladder carcinoma with SS depth of invasion. However, in cases where the hyperechoic outer layer is retained, the depth of invasion may extend to the mucous membrane, muscularis, or shallow SS layer, depending on the case, and differentiation is difficult even by EUS.

### 5. Wall-thickening lesions

#### i) Gallbladder adenomyomatosis

**Fig. 15:**
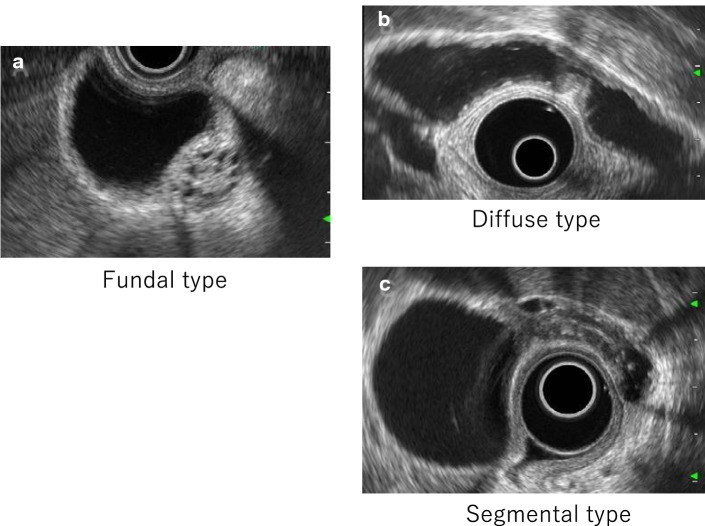
Gallbladder adenomyomatosis. **a** Fundal type. **b** Diffuse type. **c** Segmental type

Histopathologically, gallbladder adenomyomatosis is a disease that causes RAS and thickening of the gallbladder wall owing to smooth muscle and fibrous tissue hyperplasia. Based on the location and morphology of the wall lesions, gallbladder adenomyomatosis is classified as fundal (with a focal lesion involving the gallbladder’s fundal region), segmental (with thickening of the gallbladder neck or body), or diffuse (with RAS hyperplasia and thickening that involve the whole gallbladder wall) (Fig. 15).

In gallbladder adenomyomatosis, the thickened wall has a smooth surface but occasionally exhibits surface irregularity, reflecting hyperplastic changes. A key point in its diagnosis is to confirm the presence of cystic anechoic spots reflecting RAS inside the thickened wall. Comet tail artifacts are also occasionally observed owing to multipath reflection from RAS or intramural calculi.

#### ii) Xanthogranulomatous cholecystitis

Xanthogranulomatous cholecystitis is a unique form of cholecystitis in which the gallbladder wall thickening primarily involves the SS layer and is accompanied by irregular thickening of the gallbladder wall and fibrosis. As the inflammation occasionally affects surrounding organs such as the liver and transverse colon, differentiation from gallbladder carcinoma is frequently problematic. The disease may result from impaction of stones in the neck of the gallbladder or biliary leakage into the gallbladder wall owing to RAS rupture or mucosal ulceration. In cases without lithiasis, gallbladder carcinoma may be a possible cause. Differentiation between benign and malignant types based on EUS alone is frequently difficult.

#### iii) Hyperplasia of the gallbladder mucous membrane accompanying anomalous pancreaticobiliary junction

**Fig. 16:**
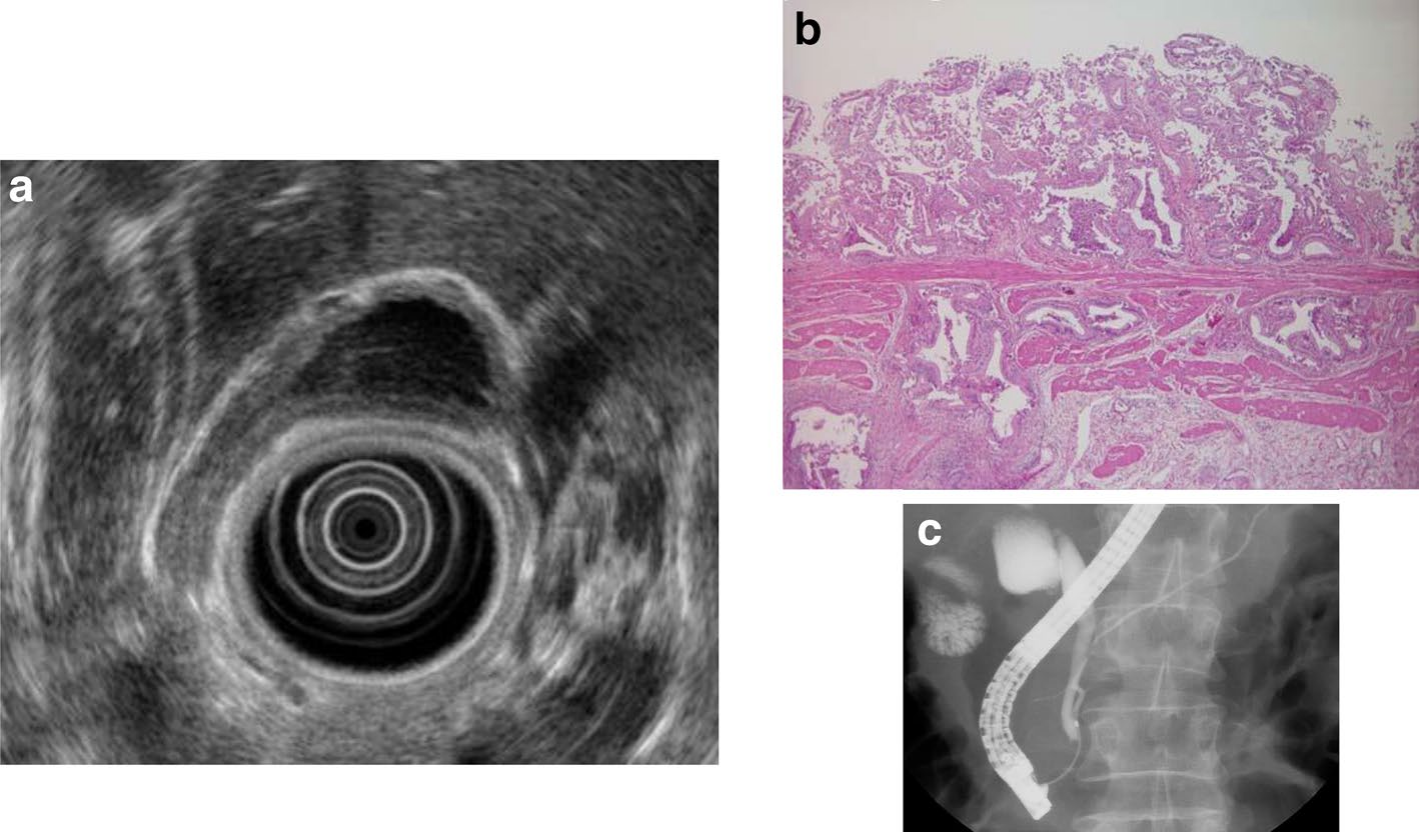
Hyperplasia of the gallbladder mucous membrane accompanying anomalous pancreaticobiliary junction. **a** EUS image of the thickened inner hypoechoic layer of the gallbladder. **b** Hyperplastic changes in the gallbladder mucous membrane. **c** ERCP image of anomalous pancreaticobiliary junction

As an anomalous pancreaticobiliary junction leads to reflux of pancreatic juice into the biliary tract, hyperplastic changes arise in the gallbladder mucous membrane (Fig. 16). Hyperplasia of the gallbladder mucous membrane is recognized in 38–63% of patients with an anomalous pancreaticobiliary junction, with an even higher rate of 90–100% particularly in patients without bile duct dilatation [21, 22].

In hyperplasia of the gallbladder mucous membrane, epithelial height is increased, cellular proliferative activity is accelerated, and a mechanism from hyperplasia to dysplasia and carcinoma is speculated.

#### iv) Gallbladder carcinoma (wall-thickening type)

**Fig. 17:**
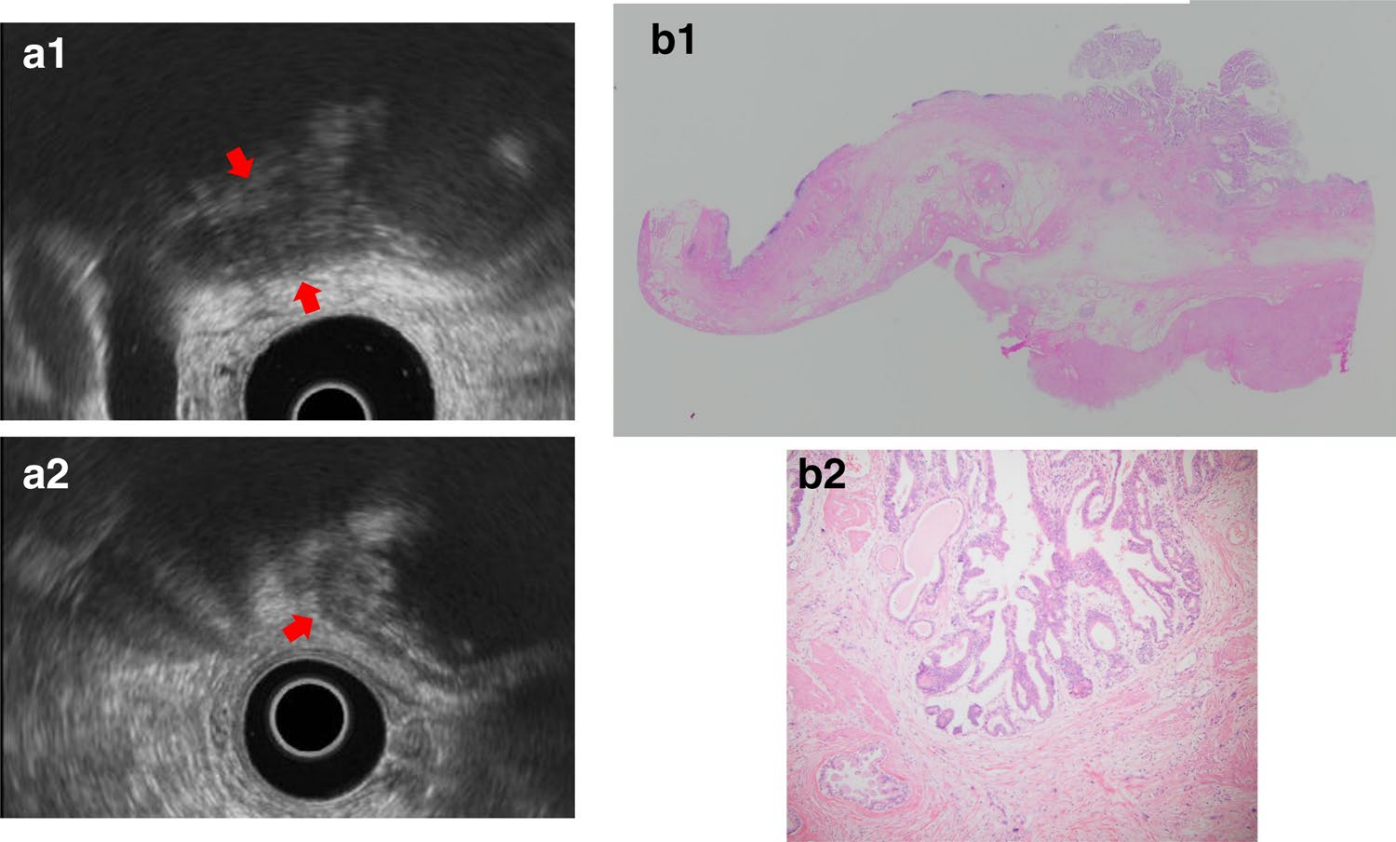
Gallbladder carcinoma (wall-thickening type). **a** (1, 2) EUS image of irregular gallbladder wall thickening from the gallbladder body to the fundus (arrow). **b** (1, 2) Photomicrograph: Gallbladder carcinoma with SS depth invasion

In the wall-thickening type, differentiation from gallbladder adenomyomatosis and chronic cholecystitis is problematic, but in gallbladder carcinoma, the mucous membrane is irregular or papillated, thickened areas do not have uniform thickness, and the layered structure is ill-defined. Furthermore, microcysts and comet tail artifacts reflecting RAS are usually not observed (Fig. 17).

### 6. EUS-FNA for gallbladder lesions

Bile duct biopsy is the first choice procedure in the pathological diagnosis of gallbladder lesions in which a biliary stricture is present. However, when a biliary stricture is absent, it is often necessary to rely on cytological examination of bile collected from the gallbladder through the cystic duct, which makes diagnosis difficult. Cytological examination using endoscopic naso-gallbladder drainage does not always have a high success rate, requires a highly proficient practitioner, and presents problematic points such as perforation of the cystic duct when using a guidewire [23–25].

Although EUS-FNA is highly useful and widely used for pancreatic carcinoma and gastrointestinal lesions, the decision to use EUS-FNA for biliary tract lesions, particularly gallbladder carcinoma, should be made with care because of risks such as biliary fistula and dissemination to membranes. Regional lymphadenopathy is often noted in unresectable advanced gallbladder carcinoma [26]. Considering the risks such as invasive biliary fistula, which may affect neighboring organs including the liver, and peritoneal dissemination, aspiration from regional lymph nodes is preferable. Hijioka et al. have reported that FNA can be performed in gallbladder lesions without compromising diagnostic performance or safety [26]. Moreover, the diagnostic performance of EUS-FNA in gallbladder lesions is high, with a sensitivity, specificity, and diagnostic accuracy of 80–100%, 100%, and 83–100%, respectively [26–31].

**Fig. 18:**
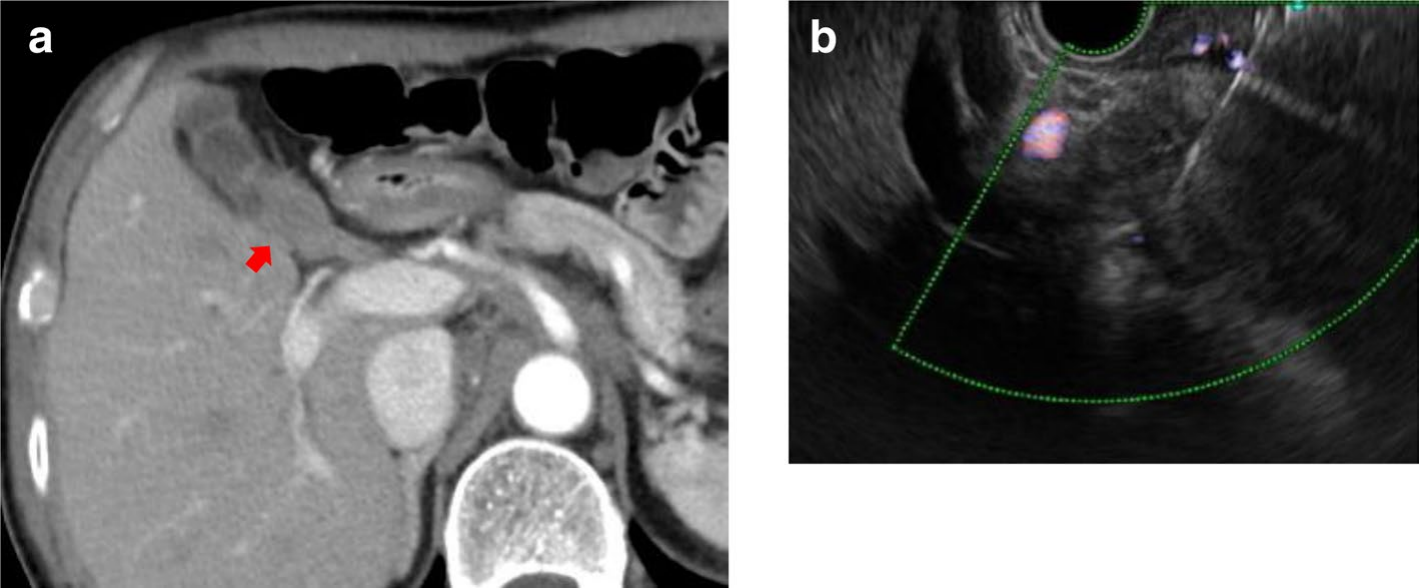
EUS-FNA for a gallbladder lesions. **a** CT scan shows a gallbladder lesion in the gallbladder neck (arrow). **b** EUS-guided FNA for a gallbladder mass lesion

When directly puncturing the gallbladder wall, despite the care taken to gain stroke distance by tangentially puncturing the gallbladder wall (Fig. 18), the wall may move if the gallbladder lumen remains and puncturing is often difficult. In cases where lesions have invaded the liver, it is recommended to puncture either the liver parenchyma as the invasion site or the gallbladder wall that is in contact with the liver parenchyma.

## Conclusion

EUS is an important testing method that plays several significant roles such as detection of gallbladder lesions, differentiation between benign and malignant types, and evaluation of malignancy progression level. In the future, the importance of EUS in this field is expected to grow further with the use and establishment of EUS-FNA for gallbladder lesions.
